# Distribution and Hospital Information System Acknowledgment Timeliness of Critical Value Reports in a Large Tertiary Hospital: A Retrospective Descriptive Study

**DOI:** 10.1155/jonm/8786244

**Published:** 2026-10-17

**Authors:** T. T. He, X. Y. Shi, S. H. Xuan, J. Zhou, H. Cong, H. M. Chen

**Affiliations:** ^1^ International Medical Center Affiliated Hospital of Nantong University Nantong Jiangsu China ahnmc.com; ^2^ Department of Laboratory Medicine Affiliated Hospital of Nantong University Nantong Jiangsu China ahnmc.com; ^3^ Department of Laboratory Medicine Affiliated Dongtai Hospital of Nantong University Dongtai Jiangsu China; ^4^ Nursing Department Affiliated Dongtai Hospital of Nantong University Dongtai Jiangsu China

**Keywords:** critical values, emergency department, HIS acknowledgment time, intensive care unit

## Abstract

**Aims:**

To describe the distribution of critical value reports, departmental patterns, and HIS‐recorded acknowledgment timeliness in a large tertiary hospital in 2023 and to identify targets for process optimization.

**Background:**

Critical value reporting is a key healthcare quality and safety process, but whole‐hospital evidence on item distribution, departmental variation, and HIS acknowledgment timeliness across high‐load settings remains limited.

**Methods:**

This retrospective, descriptive, observational study analyzed all consecutive laboratory‐related critical value records from the Affiliated Hospital of Nantong University in 2023. Records with complete timestamps were included. Variables included test item, department, visit type, specimen collection time, result dispatch time, clinical recipient, and HIS acknowledgment time. HIS acknowledgment time was defined as the interval between result dispatch/reporting and the first documented clinical acknowledgment/response in the HIS. A timely HIS acknowledgment was defined as documented acknowledgment within 15 min; a delayed HIS acknowledgment was defined as documented HIS acknowledgment requiring more than 60 min. Data were analyzed descriptively using median with interquartile range and frequency with percentage.

**Results:**

A total of 53,639 critical value records were analyzed. The highest proportions were for WBC count (21.46%), microbial culture (14.10%), PLT count (12.97%), and cTnI (12.86%). The top departments were the hematology ward (22.13%), ICU (20.65%), and emergency department (17.46%). The overall median HIS acknowledgment time was 35 s (IQR 20–96 s), and the overall timely acknowledgment rate was 94.57%. The highest untimely acknowledgment rates were observed in the emergency department (9.53%), ICU (6.98%), and hematology outpatient clinic (6.39%). The highest delayed acknowledgment rates were observed in the hematology outpatient clinic (2.51%), emergency department (2.15%), and ICU (1.01%).

**Conclusion(s):**

HIS‐recorded critical value acknowledgment was generally timely, but untimely and delayed acknowledgment clustered in specific high‐load departments. These findings can inform process optimization. Because clinical interventions and patient outcomes were not evaluated, no conclusions about patient safety outcomes can be drawn.

**Implications for Nursing Management:**

Nurse managers should ensure clear accountability for critical value acknowledgment, implement unit‐level escalation protocols, monitor item‐ and department‐level timeliness dashboards, support mobile alert access, and conduct regular audits and training, particularly in the emergency department, ICU, and hematology outpatient clinic.

## 1. Introduction

A critical value refers to the test data/result that can indicate that the patient’s life is in a dangerous/critical state, and the clinical emergency appropriate rescue measures should be taken immediately [1, 2]. The critical value reporting system serves as a cornerstone of healthcare quality and safety management, ensuring that vital information is swiftly communicated to clinical staff for prompt and effective handling. Recognized as a key performance indicator by global health authorities, this system has become increasingly vital for safeguarding patient safety. With continuous advancements in medical testing technologies and significant progress in hospital informatization, the development and optimization of critical value management systems now hold growing practical significance in maintaining patient safety standards [3–7]. Domestic and foreign guidelines and expert consensus have clearly emphasized that critical values should be notified and clinical response should be achieved within the specified time window to minimize medical risks [1, 8, 9].

Although current medical information systems have significantly improved the efficiency of critical value reporting and transmission, there remains a notable disparity in response capabilities across departments. Research indicates that these differences may be closely related to factors such as departmental workload, rationality of workflow design, staffing levels, and effectiveness of communication mechanisms [10]. As a comprehensive hospital with substantial annual patient volume, our institution generates numerous critical value reports each year, covering extensive departments and involving complex medical procedures. However, systematic evaluations of specific distribution patterns within the hospital, interdepartmental variations, and actual response efficiency among healthcare staff have yet to be conducted. Timely and effective medical responses are not only fundamental requirements for healthcare processes but also critical actions to prevent disease progression and reduce medical risks. Any delay or oversight at any stage may directly endanger patient safety, compromise treatment outcomes, or even lead to adverse medical events [8]. Therefore, an in‐depth analysis of critical value distribution patterns and departmental characteristics, along with an assessment of weak links in response workflows, holds significant practical importance for enhancing medical quality.

This study conducted a retrospective analysis of critical value data from the entire hospital throughout 2023, systematically examining the distribution patterns, departmental origins, and HIS acknowledgment timeliness of critical values. The research aims to identify existing issues in current management processes and provide data‐driven support for optimizing critical value reporting pathways and enhancing clinical response efficiency, thereby strengthening medical safety management and safeguarding patient lives.

Previous national surveys in China have described critical value reporting policies and general compliance, and recent quality‐improvement studies have evaluated closed‐loop systems or single‐department interventions. However, few studies have characterized whole‐hospital, item‐level, and department‐level HIS acknowledgment timeliness for all consecutive critical values over a full year, particularly linking high‐volume items to the departments that generate them. This study addresses that gap by analyzing 53,639 critical value records from a large tertiary hospital, including high‐load emergency and ICU settings, and by identifying actionable targets for nursing management and process redesign. Unlike single‐center quality‐improvement reports focused on a specific intervention, this is a descriptive baseline analysis of existing operational data, not an evaluation of a new closed‐loop system.

## 2. Materials and Methods

### 2.1. Study Design

This was a retrospective, descriptive, observational study conducted using laboratory‐related critical value records from a single tertiary hospital. The study was reported in accordance with the Strengthening the Reporting of Observational Studies in Epidemiology (STROBE) checklist for observational studies. A completed STROBE checklist is provided as Supporting Information (available here).

### 2.2. Study Setting and Context

The study was conducted at the Affiliated Hospital of Nantong University, a large, comprehensive tertiary hospital in Nantong, Jiangsu, China. The hospital’s Laboratory Information System (LIS) and Hospital Information System (HIS) are integrated for critical value reporting. Critical values are defined according to the hospital’s clinical laboratory guidelines and are reported directly through the HIS to clinical staff. The study period was January 1 to December 31, 2023.

### 2.3. Population and Sample

All laboratory‐related critical value records generated during the study period were eligible. Records were included if they were reported as critical values according to the hospital’s predefined critical value list and contained complete information on test item, department, specimen collection time, result dispatch time, clinical recipient, and HIS acknowledgment time. A total of 53,639 critical value records from outpatients, inpatients, and emergency department patients were collected and included in the analysis. No sampling was performed; all consecutive eligible records were analyzed. Because this was a retrospective, record‐based study, no formal sample size calculation was performed.

### 2.4. Variables and Instruments

The primary variables extracted from the LIS/HIS were as follows: test date, hospitalization number, patient visit type, department, critical value test item, specimen collection time, test result, result dispatch time, sender information, clinical recipient details, and HIS acknowledgment time. Clinical handling outcome was initially considered but was not consistently available in structured LIS/HIS fields; therefore, it was not analyzed.

Critical value items included 19 laboratory parameters: WBC, microbial culture, PLT, cTnI, GRAN, PT, K^+^, CRE, GLU, FIB, Hb, APTT, RBC, BUN, AMY, AST, Ca^++^, ALT, and Na^+^.

HIS acknowledgment time was defined as the interval between result dispatch/reporting and the first documented clinical acknowledgment/response recorded in the HIS. A timely HIS acknowledgment was defined as documented HIS acknowledgment within 15 min after dispatch. A delayed HIS acknowledgment was defined as documented HIS acknowledgment requiring more than 60 min. These measures reflect acknowledgment/documentation timeliness, not the timing of actual clinical intervention. Departments with critical value cases accounting for less than 1% of the total were grouped as “other departments” for descriptive analysis.

### 2.5. Procedure

Critical value data were retrospectively extracted from the LIS/HIS for the full year 2023. All critical values were reported according to the hospital’s clinical laboratory guidelines, using the HIS for direct reporting. The data extraction and verification process included the following steps: (1) the LIS generated a list of all critical value records during the study period; (2) two investigators independently reviewed and extracted the prespecified variables; (3) discrepancies were resolved by consensus or by a third investigator; and (4) the final dataset was deidentified before analysis. The HIS acknowledgment time and timeliness indicators were calculated from the recorded dispatch and HIS acknowledgment timestamps.

### 2.6. Data Analysis

Statistical analyses were performed using Excel (Microsoft Corporation, Redmond, WA, USA) and SPSS Version 20.0 (IBM Corp., Armonk, NY, USA). Graphs were generated using GraphPad Prism 7.0 (GraphPad Software, San Diego, CA, USA). The normality of continuous variables was assessed using the Kolmogorov–Smirnov test. HIS acknowledgment time data were not normally distributed; therefore, they were described as the median with interquartile range [*M* (IQR)]. Categorical variables were presented as frequencies and percentages [*n* (%)]. As this was a descriptive observational study, no a priori hypothesis testing was planned, and no inferential statistical tests were performed. Missing data were not imputed; records with missing key timestamps were excluded from the corresponding response‐time calculations.

The following formulas were used:

 Timely HIS acknowledgment rate = (number of critical value records with documented HIS acknowledgment within 15 min/total number of critical value records requiring acknowledgment during the same period) × 100%.
 Delayed HIS acknowledgment rate = (number of critical value records with documented HIS acknowledgment after 60 min/total number of critical value records requiring acknowledgment) × 100%.
 All rates were reported at the hospital level, by critical value item, and by department.

### 2.7. Ethical Considerations

This study was approved by the Ethics Committee of the Affiliated Hospital of Nantong University (approval number: 2022‐K057‐01). The study was conducted in accordance with the Declaration of Helsinki. Because this was a retrospective analysis of existing patient‐linked laboratory and hospital records, the Ethics Committee waived the requirement for informed consent. Patient‐linked records were accessed under the approved waiver and were deidentified before analysis. No directly identifiable personal information is reported in this manuscript.

## 3. Results

### 3.1. Overall Distribution of Critical Value Records

From January to December 2023, the hospital recorded a total of 53,639 critical value records. These critical values were categorized based on patients’ visit locations into outpatient patients, emergency department patients, and inpatients. Among them, there were 5153 critical value cases (accounting for 9.61%) among outpatient patients, 9364 critical value cases (accounting for 17.46%) among emergency department patients, and 39,122 critical value cases (accounting for 72.93%) among inpatients.

### 3.2. Distribution of Critical Value Items

Among the critical value testing items, the top five items by number and proportion were WBC, microbial cultures, PLT, cTnI, and GRAN. The first four items each accounted for over 10% of total cases. Specifically, WBC accounted for 11,513 records (21.46%), microbial cultures in 7564 cases (14.10%), PLT in 6956 cases (12.97%), and cTnI in 6899 cases (12.86%). The detailed distribution of each item is shown in Table 1.

**TABLE 1:** Distribution of critical value items.

Item	*n*	Proportion (%)
WBC	11,513	21.46
Microbial culture	7564	14.10
PLT	6956	12.97
cTnI	6899	12.86
GRAN	3211	5.99
PT	3000	5.59
K^+^	2453	4.57
CRE	2306	4.30
GLU	1624	3.03
FIB	1476	2.75
Hb	1320	2.46
APTT	1042	1.94
RBC	1025	1.91
BUN	847	1.58
AMY	770	1.44
AST	518	0.97
Ca^++^	513	0.96
ALT	384	0.72
Na^+^	218	0.41
Total	53,639	100

In terms of the distribution of critical value items in departments, the hematology ward had the highest number and proportion of WBC and PLT critical values, while the intensive care unit (ICU) had the highest number and proportion of microbial culture and cTnI critical values. The specific distribution of the four items in departments is shown in Figure 1.

**FIGURE 1:**
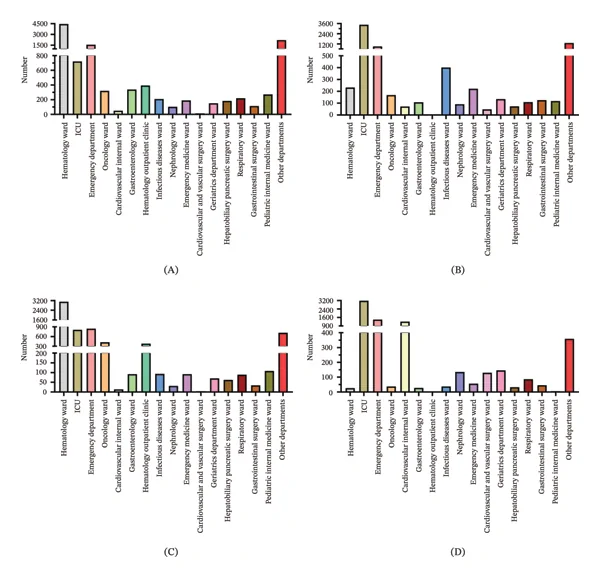
Department distribution of critical value items with a percentage greater than 10%. (A) The departmental distribution of critical values for WBC. (B) The departmental distribution of critical values for microbial culture. (C) The departmental distribution of critical values for PLT. (D) The departmental distribution of critical values for cTnI.

### 3.3. Distribution of Critical Value Departments

For statistical purposes, departments with critical value cases accounting for less than 1% were classified as “other departments”. The distribution of critical value departments across the hospital is shown in Table 2. The top five departments by number and proportion of critical value records were as follows: hematology ward, ICU, emergency department, oncology ward, and cardiovascular internal ward. Notably, the hematology ward, ICU, and emergency department each accounted for over 10% of critical value cases, with 11,871 (22.13%), 11,074 (20.65%), and 9364 (17.46%) cases, respectively.

**TABLE 2:** Distribution of critical values across departments.

Department	*n*	Proportion (%)
Hematology ward	11,871	22.13
ICU	11,074	20.65
Emergency department	9364	17.46
Oncology ward	1771	3.30
Cardiovascular internal ward	1717	3.20
Gastroenterology ward	1244	2.32
Hematology outpatient clinic	1237	2.31
Infectious diseases ward	893	1.66
Nephrology ward	854	1.59
Emergency medicine ward	738	1.38
Cardiovascular and vascular surgery ward	716	1.33
Geriatrics department ward	706	1.32
Hepatobiliary pancreatic surgery ward	684	1.28
Respiratory ward	665	1.24
Gastrointestinal surgery ward	641	1.20
Pediatric internal medicine ward	583	1.09
Other departments	8881	16.56

In the hematology ward, critical values are primarily measured by WBC, PLT, and GRAN, accounting for 37.49%, 26.12%, and 23.79% of total critical values, respectively. The ICU focuses on microbial cultures and cTnI, representing 31.27% and 28.72% of critical values, respectively. The emergency department’s key indicators include WBC (16.71%), cTnI (14.85%), and CRE (12.40%). Detailed breakdowns of critical value components exceeding 10% in each department are shown in Supporting Figure S1.

### 3.4. Critical Value Item HIS Acknowledgment Time and HIS Acknowledgment Rate

In 2023, the shortest critical value HIS acknowledgment time across the hospital was 1 s, while the longest lasted 5397 s (approximately 90 min), with a median HIS acknowledgment time of 35 s (20–96 s). A timely HIS acknowledgment was defined as documented acknowledgment within 15 min. The HIS acknowledgment rate was calculated using the formula: (Number of critical value records with documented HIS acknowledgment within 15 min/Total number of critical value records requiring acknowledgment during the same period) × 100%. Among the 53,639 critical value records, 50,725 had documented HIS acknowledgment within 15 min, achieving a total timely HIS acknowledgment rate of 94.57%. All 19 critical value items involved showed HIS acknowledgment rates above 90%, with GRAN count demonstrating the highest rate at 97.82% (median HIS acknowledgment time: 18 s) and APTT showing the lowest rate at 91.36% (median HIS acknowledgment time: 59 s). Detailed HIS acknowledgment times and timely rates for each critical value item are presented in Table 3.

**TABLE 3:** Critical value item HIS acknowledgment time and timely acknowledgment rate.

Item	HIS acknowledgment time (s), median (IQR)	Timely HIS acknowledgment rate (%)
WBC	30 (18–75)	95.15
Microbial culture	48 (29–103)	92.81
PLT	29 (18–80)	95.20
cTnI	30 (17–92)	95.81
GRAN	18 (13–34)	97.82
PT	36 (19–108)	94.90
K^+^	38 (22–118)	95.35
CRE	42 (21–149)	92.02
GLU	43 (24–132)	94.27
FIB	49 (24–160)	93.83
Hb	38 (21–109)	92.88
APTT	59 (27–223)	91.36
RBC	41 (24–105)	93.27
BUN	45 (22–163)	91.74
AMY	37 (21–118)	92.73
AST	43 (23–135)	91.51
Ca^++^	46 (25–153)	96.88
ALT	48 (26–150)	91.67
Na^+^	53 (30–140)	94.50

### 3.5. HIS Acknowledgment Time and HIS Acknowledgment Rate of Critical Values in Each Department

Among all departments, the hematology ward had the shortest median HIS acknowledgment time for critical values at 21 s, while Pediatric Internal Medicine Ward recorded the longest HIS acknowledgment time of 64 s. All departments achieved a timely HIS acknowledgment rate exceeding 90%, with the respiratory ward leading at 99.1%. The emergency department showed the lowest HIS acknowledgment rate at 90.47%. Detailed breakdowns of critical value HIS acknowledgment times and their corresponding rates across departments are provided in Table 4.

**TABLE 4:** Critical value HIS acknowledgment time and timely acknowledgment rate by department.

Department	HIS acknowledgment time (s), median (IQR)	Timely HIS acknowledgment rate (%)
Hematology ward	21 (14–39)	97.55
ICU	43 (22–139)	93.02
Emergency department	37 (20–133)	90.47
Oncology ward	35 (21–104)	95.48
Cardiovascular internal ward	26 (17–54)	98.43
Gastroenterology ward	41 (25–117)	97.35
Hematology outpatient clinic	27 (17–64)	93.61
Infectious diseases ward	50 (32–94)	95.63
Nephrology ward	37 (23–72)	98.01
Emergency medicine ward	42 (26–94)	96.88
Cardiovascular and vascular surgery ward	29 (16–87)	98.18
Geriatrics department ward	36 (24–80)	98.30
Hepatobiliary pancreatic surgery ward	37 (23–84)	97.95
Respiratory ward	31 (21–62)	99.10
Gastrointestinal surgery ward	44 (27–104)	97.50
Pediatric internal medicine ward	64 (38–134)	98.97
Other departments	51 (28–148)	93.32

### 3.6. Untimely and Delayed HIS Acknowledgment Rates of Critical Values in Each Department

A total of 2914 critical value notifications had no documented HIS acknowledgment within 15 min, giving an untimely acknowledgment rate of 5.43%. Departments with the highest rates were the emergency department (9.53%, 892 cases), ICU (6.98%, 773 cases), and hematology outpatient clinic (6.39%, 79 cases). A case was defined as “delayed HIS acknowledgment” when documented HIS acknowledgment occurred more than 60 min after dispatch. The delayed HIS acknowledgment rate was calculated as follows: (number of critical value records with documented HIS acknowledgment after 60 min/total critical value records requiring acknowledgment) × 100%. Among all records, 536 had documented HIS acknowledgment after 60 min, giving a delayed acknowledgment rate of 1.00%. Departments with higher delayed HIS acknowledgment rates included hematology outpatient clinic (2.51%, 31 cases), emergency department (2.15%, 201 cases), and ICU (1.01%, 112 cases). Detailed data on untimely and delayed HIS acknowledgment rates across departments are presented in Table 5.

**TABLE 5:** The untimely HIS acknowledgment rate and delayed HIS acknowledgment rate of critical values in various departments.

Department	Critical value count	No documented HIS acknowledgment within 15 min		Delayed HIS acknowledgment (> 60 min)	
		*n*	%	*n*	%
Hematology ward	11,871	291	2.45	37	0.31
ICU	11,074	773	6.98	112	1.01
Emergency department	9364	892	9.53	201	2.15
Oncology ward	1771	80	4.52	10	0.56
Cardiovascular internal ward	1717	27	1.57	3	0.17
Gastroenterology ward	1244	33	2.65	3	0.24
Hematology outpatient clinic	1237	79	6.39	31	2.51
Infectious diseases ward	893	39	4.37	3	0.34
Nephrology ward	854	17	1.99	3	0.35
Emergency medicine ward	738	23	3.12	2	0.27
Cardiovascular and vascular surgery ward	716	13	1.82	1	0.14
Geriatrics department ward	706	12	1.7	3	0.42
Hepatobiliary pancreatic surgery ward	684	14	2.05	1	0.15
Respiratory ward	665	6	0.9	0	0
Gastrointestinal surgery ward	641	16	2.5	0	0
Pediatric internal medicine ward	583	6	1.03	0	0
Other departments	8881	593	6.68	126	1.42
Total	53,639	2914		536	

## 4. Discussion

This study conducted a retrospective analysis of 53,639 critical value reports from our hospital in 2023, revealing distribution patterns of critical values, departmental characteristics, and HIS acknowledgment timeliness. Although preliminary observations indicated satisfactory overall response efficiency, the findings highlighted several key areas requiring focused attention. In this discussion, we will interpret critical findings, conduct comparative analyses with existing literature, explore potential systemic issues in depth, and propose concrete strategies for optimizing critical value notification processes.

A higher proportion of inpatient departments in critical value (72.93%) was expected, as hospitalized patients typically have more severe conditions and require closer monitoring. This finding is consistent with the results of previous large‐scale critical value practice surveys in China [10]. The relatively lower proportion of emergency departments and outpatient departments reflects differences in patient severity across different medical settings. Among critical value indicators, WBC (21.46%), microbial culture (14.10%), PLT (12.97%), and cTnI (12.86%) collectively accounted for over 60% of all critical values. This distribution is not random but reflects the clinical value of these laboratory parameters. A sharp elevation or decrease in WBC levels can directly indicate severe infections (e.g., sepsis), hematologic disorders (e.g., leukemia), or immunosuppressed states [11]. A significant reduction in PLT counts increases the risk of life‐threatening hemorrhage, commonly observed in hematologic diseases, postchemotherapy conditions, disseminated intravascular coagulation (DIC), and severe infections [12]. Positive cultures for drug‐resistant bacteria or highly virulent pathogens (particularly from blood or cerebrospinal fluid specimens) remain the gold standard for diagnosing invasive infections, bacteremia, or sepsis [13]. Elevated cTnI levels are characteristic markers of critical conditions such as acute myocardial infarction, heart failure, pulmonary embolism, severe infections, and renal dysfunction, making them pivotal parameters for assessing cardiac involvement and prognosis [14].

The differences in distribution patterns across various departments reflect the characteristics of the underlying disease spectra in each specialty. In hematology wards, key indicators primarily include WBC, PLT, and GRAN. This finding aligns with the pathophysiological mechanisms of hematologic malignancies and the bone marrow suppression effects of chemotherapy. In the ICU, microbial cultures and cTnI are predominant, reflecting the high incidence of nosocomial infections and cardiac complications in critically ill patients [13, 14]. The distribution characteristics in the emergency department exhibit greater heterogeneity, with WBC, cTnI, and CRE serving as primary indicators, highlighting the diversity of acute conditions admitted to this department [15].

The hospital’s overall timely HIS acknowledgment rate was 94.57%, comparable to data previously reported in China [10]. 5.43% of critical value notifications had no documented HIS acknowledgment within 15 min, and 1.00% had no documented HIS acknowledgment within 60 min. This does not establish that no clinical intervention occurred; interventions may have been delivered without timely HIS documentation. These findings should, therefore, be interpreted as process and documentation timeliness indicators rather than as direct measures of treatment delay. Several potential explanations may account for the observed departmental differences, although these factors were not measured in this study. Emergency Department and ICU staff work in alarm‐rich environments; it is plausible that alarm burden and competing urgent tasks could contribute to delayed HIS acknowledgment. Peak‐hour workload and patient flow may also affect acknowledgment, but staffing ratios and workload were not quantified. In the hematology outpatient clinic, high patient volume and staff turnover could be relevant, but this remains hypothesis‐generating. Shared responsibility among multiple providers might dilute accountability, but team structure and notification responsibility were not measured. These possible mechanisms should be examined in future studies with direct measures of workload, staffing, alarm burden, and workflow. In addition, although our current definition of “timely response” (15 min) is consistent with the Chinese national guidelines [1], it may not be stringent enough for certain critical conditions. For example, in cases of suspected sepsis or acute ST‐segment elevation myocardial infarction, the guidelines recommend antibiotic therapy or reperfusion therapy within 30–60 min after presentation. If there is a 15‐min delay in confirming the critical threshold before initiating treatment, it may indicate a clinically significant proportion of time within the therapeutic window. Future revisions to the critical threshold policy should be based on specific testing methods and clinical scenarios, considering the adoption of risk‐stratified time thresholds.

Based on the findings of this study, the following concrete, evidence‐informed measures are proposed to improve critical value response efficiency, particularly in high‐risk departments: (1) The risk stratification notification algorithm is employed. When the numerical value exceeds the preset critical threshold range (e.g., cTnI > 10 times the upper limit of the normal reference range), more prominent visual and auditory alarms are triggered to distinguish it from the critical value boundary. (2) By leveraging the integration capabilities of mobile workstations, it ensures that critical value notifications are delivered in real‐time to bedside devices used during resuscitation or trauma care (such as handheld phones, wearable devices, or tablet workstations), preventing healthcare professionals from missing important alerts when they are away from fixed computers. (3) If conditions permit, a dedicated critical value coordinator can be appointed: during peak hours, a designated nurse or physician should serve as the critical value coordinator to receive and record notification information, ensuring accountability implementation. In the ICU, filter out nonimmediate alerts by collaborating with the clinical engineering department to suppress or downgrade routine equipment alerts that do not require immediate attention. This approach reduces overall alert load and enhances the significance of critical value notifications. An automated reporting mechanism should be established. If the responsible nurse fails to confirm the critical value within 5 min, the system will automatically report the notification to the attending physician; if confirmation is still absent after 10 min, the report will be escalated to the department head. For outpatients, telephone reporting of critical values is particularly crucial. Additionally, during registration, a contact phone number must be retained for each outpatient to ensure immediate notification via phone if the attending physician is unavailable. For cases with delayed HIS acknowledgments (exceeding 60 min), the hospital will conduct weekly audits and provide feedback to all departments. Concurrently, existing information systems must be upgraded to comprehensively record critical values from physician review time, order issuance time, and treatment implementation time, thereby enabling automated identification of specific bottleneck processes rather than merely maintaining basic confirmation levels.

The overall timely HIS acknowledgment rate of our hospital was 94.57%, higher than the range of 85.3%–91.2% reported in a nationwide survey conducted in 2017 involving 973 Chinese laboratories [10], indicating improved hospital informatization levels and awareness among medical staff. However, direct comparisons should be made with caution due to differences in study duration, hospital characteristics, and definitions of “timeliness.” The median HIS acknowledgment time observed in this study was 35 s (IQR: 20–96 s), significantly shorter than previously reported critical threshold notification turnaround times [7, 9]. Nevertheless, the presence of outliers with HIS acknowledgment times exceeding 90 min in our hospital suggests that system resilience under peak loads still requires improvement.

This study has some limitations. Firstly, the single‐center study design limits the generalizability of findings to other hospital settings, which may differ in patient demographics, staffing levels, information systems, and critical value management policies. Secondly, although the study identified response delays and delayed HIS acknowledgments, the lack of associated patient outcome data prevented reliable assessment of the clinical consequences of these delays (e.g., adverse events, prolonged hospital stays, disability, or mortality). Thirdly, this analysis did not account for several potential confounding factors, including individualized patient assessment indicators (e.g., triage level and APACHE score); shift differences (night shifts vs. day shifts); clinical physician characteristics (years of practice, specialty training background, and prior experience with critical value response procedures); and concurrent diagnostic needs at notification time (e.g., requiring simultaneous management of other critically ill patients). Fourthly, the study adopted a uniform 15 min standard for “timely response” across all 19 test types. However, recent evidence suggests that certain critical values, such as positive blood culture results in suspected sepsis or life‐threatening electrolyte disturbances, may require shorter HIS acknowledgment time windows. Future studies should comprehensively evaluate the complete process from notification to clinical intervention and patient outcomes.

## 5. Conclusion

In conclusion, HIS‐recorded acknowledgment of critical values was generally timely, but untimely and delayed acknowledgment clustered in specific items and high‐load departments. These findings can inform process optimization and nursing management. Because clinical interventions and patient outcomes were not evaluated, no conclusions can be drawn about patient safety outcomes.

## 6. Implications for Nursing Management

Nurse managers should assign clear responsibility for critical value acknowledgment during each shift, use simple escalation rules (5 min: nurse in charge; 10 min: attending physician; 15 min: department head), and monitor unit‐level timeliness dashboards, especially in the emergency department, ICU, and hematology outpatient clinic. They should also support mobile alerts, reduce nonurgent alarms, maintain reliable outpatient telephone contacts, and provide regular training and nonpunitive audit feedback. These actions target process timeliness; their effect on patient outcomes requires further study.

## Funding

This research received no specific grant from any funding agency in the public, commercial, or not‐for‐profit sectors.

## Conflicts of Interest

The authors declare no conflicts of interest.

## Supporting Information

Additional supporting information can be found online in the Supporting Information section.

## Supporting information


**Supporting Information** Supporting Figure S1 presents the departmental distribution of critical value items in the hematology ward (A), ICU (B), and emergency department (C), providing department‐level detail for the highest volume critical value items.

## Data Availability

The deidentified datasets generated and analyzed during the current study are available from the corresponding author on reasonable request, subject to institutional data governance approval.
