# Supplementary material for: Distribution and Hospital Information System Acknowledgment Timeliness of Critical Value Reports in a Large Tertiary Hospital: A Retrospective Descriptive Study
Source: J Nurs Manag. 2026 Oct 17;2026:8786244. doi: 10.1155/jonm/8786244 (PMC13676303; doi:10.1155/jonm/8786244)
Supplement: Supplementary file 1 [file JONM-2026-8786244-s001.doc]

**SUPPLEMENTARY FIGURE S1** Distribution of critical value items in high-volume departments. A. Hematology Ward. B. ICU. C. Emergency Department.
